# Cellular and Humoral Immunogenicity Investigation of Single and Repeated Allogeneic Tenogenic Primed Mesenchymal Stem Cell Treatments in Horses Suffering From Tendon Injuries

**DOI:** 10.3389/fvets.2021.789293

**Published:** 2022-02-24

**Authors:** Eva Depuydt, Sarah Y. Broeckx, Koen Chiers, Marco Patruno, Laura Da Dalt, Luc Duchateau, Jimmy Saunders, Frederik Pille, Ann Martens, Lore Van Hecke, Jan H. Spaas

**Affiliations:** ^1^Boehringer Ingelheim Veterinary Medicine Belgium, Evergem, Belgium; ^2^Department of Surgery and Anaesthesiology of Domestic Animals, Faculty of Veterinary Medicine, Ghent University, Merelbeke, Belgium; ^3^Department of Pathology, Bacteriology and Poultry Diseases, Faculty of Veterinary Medicine, Ghent University, Merelbeke, Belgium; ^4^Department of Comparative Biomedicine and Food Science (BCA), University of Padova, Padova, Italy; ^5^Biometrics Research Group, Faculty of Veterinary Medicine, Ghent University, Merelbeke, Belgium; ^6^Department of Veterinary Medical Imaging and Small Animal Orthopaedics, Faculty of Veterinary Medicine, Ghent University, Merelbeke, Belgium

**Keywords:** allogeneic, equine mesenchymal stem cell, immunogenicity, alloantibody, tendon, mixed lymphocyte reaction

## Abstract

The use of mesenchymal stem cells (MSCs) for the treatment of equine tendon disease is widely investigated because of their regenerative and immunomodulatory potential. However, questions have been raised concerning the immunogenic properties of allogeneic MSCs. Therefore, two studies were conducted to assess the safety of equine allogeneic peripheral blood-derived tenogenic primed MSCs (tpMSCs). The objective was to evaluate if a single and repeated tpMSC administration induced a cellular and humoral immune response in horses suffering from tendon injuries. Horses enrolled in the first study (*n* = 8) had a surgically induced superficial digital flexor tendon core lesion and were treated intralesionally with tpMSCs. Before and after treatment the cellular immunogenicity was assessed by modified mixed lymphocyte reactions. The humoral immune response was investigated using a crossmatch assay. Presence of anti-bovine serum albumin (BSA) antibodies was detected via ELISA. Horses enrolled in the second study (*n* = 6) suffered from a naturally occurring tendon injury and were treated twice with tpMSCs. Blood was collected after the second treatment for the same immunological assays. No cellular immune response was found in any of the horses. One out of eight horses in the first study and none of the horses in the second study had anti-tpMSC antibodies. This particular horse had an equine sarcoid and further investigation revealed presence of antibodies against sarcoid cells and epithelial-like stem cells before treatment, which increased after treatment. Additionally, formation of antibodies against BSA was observed. These findings might indicate a non-specific immune response generated after treatment. Serum from the other horses revealed no such antibody formation. These two studies showed that the administration of tpMSCs did not induce a cellular or humoral immune response following an intralesional single or repeated (two consecutive) allogeneic tpMSC treatment in horses with tendon injury, except for one horse. Therefore, a larger field study should confirm these findings and support the safe use of tpMSCs as a therapeutic for horses suffering from tendon injuries.

## Introduction

Autologous equine mesenchymal stem cells (MSCs) are widely used in clinical studies as regenerative treatment for musculoskeletal diseases in horses (1–5). Harvesting and cultivating autologous MSCs is an invasive, expensive and time-consuming process with disadvantageous effects of donor age and disease state on MSC functionality (6–10). Therefore, recent research focusses on allogeneic treatments offering an off-the-shelf, quality controlled, standardized MSC product (11–16). The use of MSCs for the treatment of equine tendon disease is investigated because of their regenerative and immunomodulatory potential. However, their tenogenic properties and regenerative effects could be compromised by the inflammatory environment of the acute tendon injury (17). Moreover, although not in horses, ectopic bone formation has been reported in mice and rabbits after administration of bone marrow-derived MSCs (BM-MSCs) into acute tendon lesions (18–21). In order to overcome these disadvantageous effects, MSCs can be tenogenic primed before clinical application (22). Our research group has administered allogeneic tenogenic primed and chondrogenic induced MSCs in horses suffering from tendon (23–25) and joint (26–29) injuries respectively, reporting minimal local adverse effects. However, controversy exists on the immunogenic properties and thus the safe use of allogeneic MSC treatments (30, 31). The induction of the humoral and cellular immune responses after major histocompatibility complex (MHC)-mismatched MSC treatment has been described (32–34) and could result in potential immunological abnormalities following repeated administration. Because it has been reported that priming the MSCs could induce a higher MHC-I and MHC-II expression (34, 35), the immunogenicity of allogeneic tenogenic primed MSCs should be investigated, emphasizing the importance of current research.

The most widely used *in vitro* test to investigate the cellular immunogenicity of MSCs is the mixed lymphocyte reaction (MLR) (36). Previously published MLR results in horses of allogeneic BM-MSCs co-cultured with equine peripheral blood-derived mononuclear cells (PBMCs) described no induction of the host's cellular immune response (13, 37). However, Schnabel et al., reported that MHC-II positive BM-MSCs cause significantly increased responder PBMC proliferation and that the immunogenicity of MHC-II negative MSC treatments should also be taken under consideration (32). For this reason, the immunogenicity of an allogeneic MSC treatment should be taken under consideration when evaluating MSC treatment options (38, 39).

In addition to a cellular immune response, administration of allogeneic MSCs could result in a humoral immune response by the recipient. Antibody production following an allogeneic MSC administration has been reported in the horse (15, 33, 34, 40, 41). The possible production of anti-MSC antibodies must be taken under consideration when performing repeated MSC injections. Although repeated injection of allogeneic MSCs has already been described in dogs (42) and horses (34, 41, 43), few studies have investigated the presence of anti-MSC antibodies (15, 33, 34, 42). Therefore, the current study evaluates the cellular and humoral response against a single and a repeated dose of equine allogeneic peripheral blood-derived tenogenic primed MSCs in horses with tendon injuries.

Another aspect of an immunological concern to consider is that a (semi)annual vaccination of horses [against equine influenza, rhinopneumonitis and tetanus for example (44)] can lead to the induction of an immune response to xenoproteins present in the vaccine. Most viral vaccines contain bovine serum albumin (BSA), the major component of fetal bovine serum (FBS), which can result in the development of anti-BSA antibodies (45). Because the tpMSCs are cultured in medium supplemented with FBS, it is important to correctly differentiate between antibody responses directed toward the tpMSCs and those directed toward a xenoprotein in which the tpMSCs are cultivated. Therefore, the detection of anti-BSA antibodies is also included in the current immunogenicity investigation.

The objective of this investigation is to evaluate the cellular and humoral immunogenicity of a single and repeated intralesional injection of equine allogeneic tenogenic primed peripheral blood-derived MSCs (tpMSCs) in horses suffering from tendon injuries.

## Materials and Methods

### Horses

The blood collection from the horses (EC_2017_001) including the blood collection from the donor horses (EC_2016_003) was approved by an independent ethics committee approved by the Flemish Government (permit number: LA1700607). The research of the present study was in accordance with national and international animal welfare regulations (Directive 2001/82/EC as amended, Belgian animal welfare legislation (KB 29/05/2013), Directive 2010/63/EU and EMEA/CVMP/816/00-Final).

For the first study, eight warmblood horses (four geldings and four mares; age ranged between 3 and 12 years) with a surgically induced superficial digital flexor tendon (SDFT) core lesion enrolled in a previous study performed by our group received a single intralesional treatment with a proprietary formulation of 3 × 10^6^ equine allogeneic tenogenic primed mesenchymal stem cells (tpMSCs) (46). To assess the cellular immune response, 9 mL of whole blood was collected aseptically from the *vena jugularis* into sterile ethylenediaminetetraacetic acid (EDTA) tubes before treatment and 16 weeks after treatment. For the investigation of the humoral immune response, blood was collected before and 2 weeks after tpMSC administration in serum clot activator blood collection tubes.

For the second study, blood was collected 6 to 8 weeks after the second tpMSC treatment from six client-owned warmblood horses (two geldings, two mares and two stallions; age ranged between 8 and 19 years) suffering from naturally occurring tendon disease receiving a repeated intralesional tpMSC administration (time range of 7 to 9 weeks between the two tpMSC administrations). The treatment consisted of 3 × 10^6^ tpMSCs for each injection. From these horses, peripheral blood was collected on a single occasion to reduce inter-test variation.

### Isolation and Tenogenic Priming of Mesenchymal Stem Cells

Tenogenic primed MSCs were prepared from the peripheral blood from one adult donor horse and characterized as previously described (23, 47). Briefly, blood was collected aseptically from the *vena jugularis* in sterile EDTA tubes. After gradient centrifugation, the interphase was isolated, seeded and cultivated in culture medium [Dulbecco's modified Eagle medium low glucose (DMEM; Life Technologies) supplemented with FBS (Sigma-Aldrich)]. Subsequently, the MSCs were cultured until passage 5 and thoroughly characterized (i.e. viability, morphology, presence of cell surface markers, and trilineage differentiation) as previously described (47), prior of being frozen as intermediate cell stock. After characterization, cells were thawed, further cultivated and tenogenic primed by adding tenogenic growth factors to the culture medium. The specific growth factors and the markers used for characterization of the tenogenic priming cannot be disclosed due to company policies. At 80% confluency, the cells were trypsinized and resuspended at a concentration of 3 × 10^6^ tpMSCs in 1 mL DMEM supplemented with 10% dimethyl sulfoxide (Sigma-Aldrich). The samples were cryopreserved at −80°C until further use. For each treatment, tpMSCs were thawed, drawn into a syringe and injected intralesionally immediately after thawing. The donor horse was screened for equine pathogens as previously reported by our group (23) at Böse laboratory (Harsum, Germany).

### Immunophenotyping of MSCs Using Flow Cytometry

To perform an immunophenotypic characterization of the MSCs, the expression of several markers was evaluated simultaneously by flow cytometry as previously described (47). Per series, MSCs were labeled using the following panel of primary antibodies: CD29-APC (clone TS2/16, Biolegend), CD44-FITC (clone CVS18, Bio-Rad), CD90 (clone DH24A, A&E Scientific), CD45-PE-Cy5.5 (clone F10-89-4, Bio-Rad) and MHC-II-PE (clone CVS20, Bio-Rad). For the CD90 detection, a secondary anti-mouse IgM-PE-Cy7 antibody was used (clone RMM-1, Biolegend). A minimum of 10,000 counts were acquired using a BD FACSCanto II flow cytometer (BD Biosciences). In addition, cells were incubated in parallel with or without isotype-specific murine IgG1-APC (clone TS2/16, Biolegend), IgG1-FITC (clone MOPC-21, Biolegend), IgM-PE-Cy7 (clone RMM-1, Biolegend), IgG2a-PE-Cy5.5 (clone MOPC-173) and IgG1-PE (clone MOPC-21, Biolegend) to establish the background signal.

### Modified Mixed Lymphocyte Reaction

Whole blood was centrifuged, and the buffy coat was collected and diluted in Hank's balanced salt solution (HBSS, Life Technologies). Next, the suspension was layered upon an equal volume of Percoll and the interphase was collected after gradient centrifugation. PBMCs were washed and resuspended in HBSS to a concentration of 1 × 10^6^ PBMCs/mL. Subsequently, the PBMCs were labeled with carboxyfluorescein succinimidyl ester (Life Technologies) according to manufacturer's instructions to evaluate cell proliferation. Finally, PBMCs were diluted in culture medium [DMEM supplemented with FBS (Sigma-Aldrich), antibiotics/antimycotics (Sigma-Aldrich) and β-mercapto-ethanol (Sigma-Aldrich)] to a final concentration of 2 × 10^6^ cells/mL. Then, 100 μL was added to the designated wells of the u-bottom 96-well tissue-culture plate (= 2 × 10^5^ PBMCs/well). For the co-incubation samples, tpMSCs were thawed, washed and resuspended in culture medium to a final concentration of 2 × 10^5^ tpMSCs/mL. The tpMSCs were plated at a 1:10 tpMSC:PMBC ratio (16, 39). The included negative control consisted of a PBMC culture from each horse (to assess baseline PBMC proliferation). As a positive control, PBMCs from each horse were stimulated with the mitogen concanavalin A (5 μg/mL; Sigma-Aldrich) (39, 48). Cultures were maintained for 4 days in a humidified incubator at 37°C and 5% CO_2_.

After the incubation period, PBMCs were collected, stained with 7-aminoactinomycine D (7-AAD; 1:100; BioLegend) for discrimination of live and dead cells. In the viable population, the PBMC proliferation was analyzed using a flow cytometer (BD FACSCanto II, BD Biosciences).

### Flow Cytometric Crossmatch Assay

Whole blood was allowed to clot at room temperature for at least two h. Following centrifugation, serum was collected, heat-inactivated at 56°C for 30 min and cryopreserved at −20°C until further analysis.

The FCCA was adapted to previously described methods (15, 49). Briefly, the vials containing the tpMSCs were thawed, counted, suspended to a suitable concentration and blocked with goat serum to decrease non-specific antibody binding. The tpMSCs were mixed with the heat-inactivated recipient serum and stained after centrifugation using a fluorescent secondary antibody (Alexa Fluor 647 goat anti-horse IgG, 1:100; Jackson ImmunoResearch) targeting horse IgG antibodies bound to the tpMSCs. The assay negative control consisted of tpMSCs incubated with only the secondary antibody. As a positive control, tpMSCs were incubated with heat-inactivated serum derived form a hyperimmunized horse with a known high anti-MSC antibody binding before staining with the secondary antibody. After incubation, the samples were centrifuged, resuspended in flow stain buffer and stained with 7-AAD for flow cytometric analysis. The hyperimmunized horse suffered from multiple naturally occurred musculoskeletal diseases and was repeatedly treated intralesionally, intra-articularly and systemically (*vena jugularis*) with a minimal treatment interval of 2 weeks, resulting in increased IgG antibody levels. Therefore, the serum derived from this horse could be used as a positive control for the current assay.

Based on the results of the previous analysis, this protocol was also executed on the serum derived from the eight horses with a surgically induced SDFT lesion to detect alloantibodies against equine epithelial-like stem cells [EpSCs (50)] and commercially available equine sarcoid cells (E42-02/R; Friedrich-Loeffer-Institut) before and two weeks after a single tpMSC treatment (Study 1).

The alloantibody response was considered positive when an antibody binding of >15% was observed (15).

### Anti-BSA Antibody ELISA

This assay was performed as previously described (15, 49). Briefly, a 96-well ELISA plate (PerkinElmer Life and Analytical Science) was coated with 100 μL BSA (1 μg/well; Sigma-Aldrich) in a carbonate-bicarbonate buffer (63.5 mM carbonate, pH 9.6) overnight at 4°C. Then 100 μL of 1% rabbit serum albumin (Sigma-Aldrich) in phosphate buffered saline (PBS; Sigma-Aldrich) was added to block. After incubation at 37°C for 1 h, wells were washed with PBS-Tween (Sigma-Aldrich) once for 10 min and then 6 times briefly. Then, 100 μL of test serum (diluted 1:100 in wash buffer) was added to each well. Each sample was plated in duplicate. Known negative (PBS and fetal horse serum) and high positive samples (horse with a known high anti-BSA antibody binding) were run as assay controls. Plates were incubated at 37°C for 1 h and washed as described above. Next, 100 μL of rabbit anti-equine IgG H&L-HRP (diluted 1:100,000; Abcam) was added to each well. Plates were incubated at 37°C for 1 h and subsequently washed as described above. A colorimetric reaction was initiated by adding 100 μL of TMB peroxidase substrate (KPL) to each well. Plates were incubated at room temperature in the dark. The colorimetric reaction was stopped by adding 100 μL of 2N H_2_SO_4_ (Sigma-Aldrich). The plates were read at 450 nm on a microplate reader (Victor 2030 Reader, PerkinElmer Life and Analytical Science). Fold increase in color relative to the negative control was determined for each sample. Samples were considered negative for anti-BSA antibodies if they displayed a fold increase < 2 compared to the negative control (15).

### Determination of Bovine Papillomavirus (BPV)

BPV is a well-known pathogen associated with the development of equine sarcoids (11). To check if the commercially available sarcoid cell-line is correctly positive for BPV and to evaluate a potential relationship between the elevated antibody response and the presence of BPV on the EpSCs and MSCs, a PCR was performed on the sarcoid cell line, EpSC line and MSCs derived from the tpMSC donor to evaluate for presence of BPV-1 and BPV-2 DNA. DNA was extracted from MSCs, equine EpSCs and equine sarcoid cells. Samples were thawed, supernatant was removed after centrifugation and the pellet was resuspended in PBS (Sigma-Aldrich). Subsequently, cells were lysed using proteinase K and lysis buffer (DNeasy Blood and Tissue Kit; Qiagen) and DNA was extracted using a commercially available kit (DNeasy Blood and Tissue Kit; Qiagen). After extraction, DNA was mixed with equine specific primers and probes (Table 1) and iQ Supermix (Bio-Rad), after which polymerase chain reaction (PCR) was performed. The primers needed to amplify BPV are BPV+1 and BPV-1. The specific probes BPV type 1 and BPV type 2 are necessary for the detection of BPV-1 and/or BPV-2. Gene expression was analyzed on the CFX96 Real-time PCR Detection System (Bio-Rad). Interferon beta (IFN-β) was used as an internal control (house-keeping gene). The primers needed to amplify IFN-β are IFN-β-F and IFN-β-R (Table 1). The specific probe IFN-β allows detection of the IFN-β gene.

**Table 1:** Sequences and accession numbers of BPV-1, BPV-2 and IFN-β primers and probes.

**Gene**	**Sequence**	**Accession number**
BPV+1 primer	F: AATCGGGTGAGCAACCTTT	X02346
BPV-1 primer	R: TGCTGTCTCCATCCTCTTCA	M20219
BPV type 1 probe	(FAM)CGTCAATCAGGTCTAAACGCCC(BHQ1)	M14546
BPV type 2 probe	(TEXAS RED)TCAACCAGGTCTAAGCGCCC(BHQ2)	M20219
IFN-β-F primer	F: AGGTGGATCCTCCCAATGG	M14546
IFN-β-R primer	R: CGAAGCAAGTCATAGTTCACAGAAA	M14546
IFN-β probe	(FAM)CCTGCTGTGTTTCTCCACCACGGC (BHQ1)	M14546

### Statistical Analysis

#### Mixed Lymphocyte Reactions

##### Study 1 (Single tpMSC Treatment)

The fold change of each coculture/positive control was compared to the negative control. Therefore, the negative control was set to 1 and a mixed model was fitted. The Bonferroni correction for multiple comparisons was applied, setting the significance level at 0.025 (0.05/2).

##### Study 2 (Repeated tpMSC Treatment)

The normal distribution of the data was checked using the Shapiro-Wilk test. Normally distributed data were analyzed using the independent samples *T*-Test. *P*-values < 0.05 were considered statistically significant.

##### Flow Cytometric Crossmatch Assay

The normal distribution of the data was checked using the Shapiro-Wilk test. Normally distributed data were analyzed using the paired samples *T-*Test and not normally distributed data by the Wilcoxon Signed Rank Test. *P*-values < 0.05 were considered statistically significant.

## Results

### Isolation and Characterization of MSCs

The cells displayed all properties to be characterized as MSCs (51). Briefly, they were plastic-adherent, trilineage differentiation towards osteoblasts, chondroblasts and adipocytes was successful and MSCs were positive for CD29 (100%), CD44 (100%), CD90 (100%) and negative for CD45 (1%) and MHC-II (0%) (Supplementary Materials 1–3).

### Modified Mixed Lymphocyte Reaction

#### Study 1: Single tpMSC Treatment

**Figure 1:**
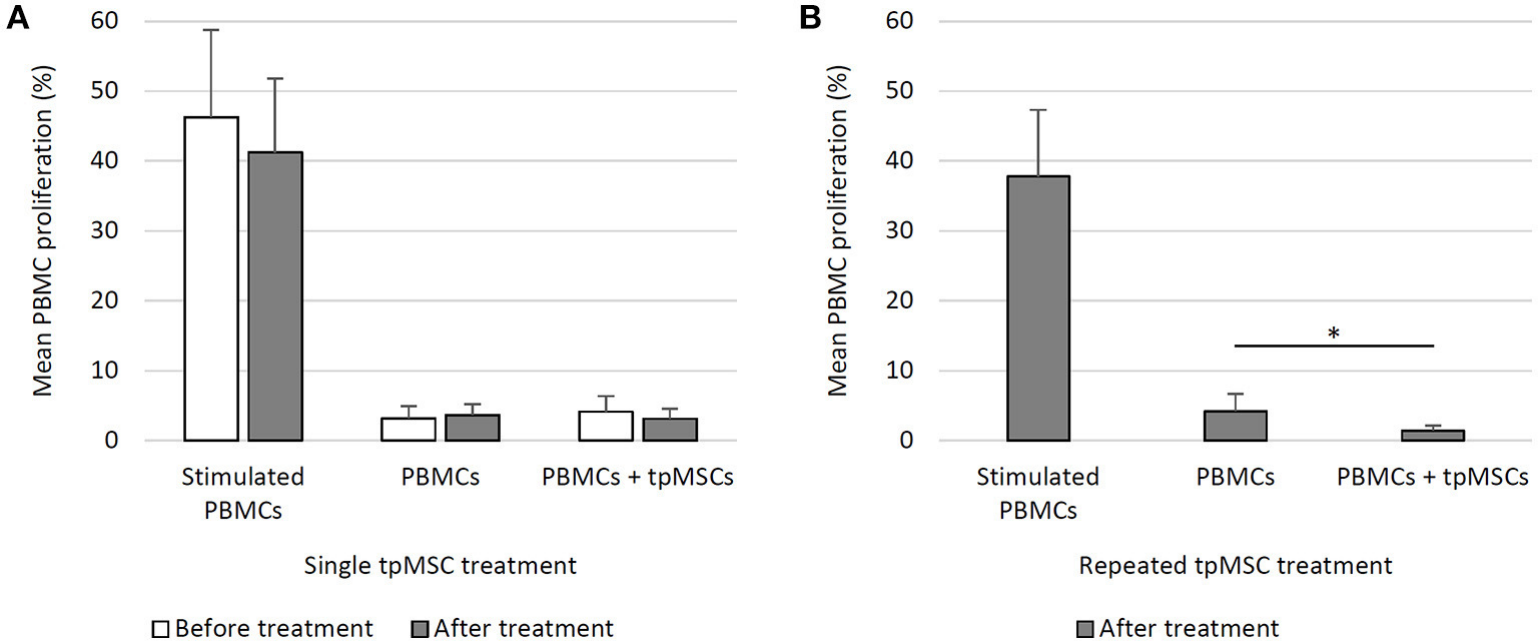
Mixed lymphocyte reaction assay. **(A)** In eight horses before and after a single tpMSC treatment, the tpMSC-PBMC coculture did not give rise to a significant increase in mean PBMC proliferation before and after a single tpMSC treatment compared to the negative control. No statistically significant difference was present between PBMC proliferation before and after treatment. **(B)** In six horses after a second tpMSC treatment, the mean PBMC proliferation percentage was significantly lower than the proliferation rate of the negative control samples. PBMCs, peripheral blood mononuclear cells; tpMSCs = tenogenic primed mesenchymal stem cells. ^*^P-values ≤ 0.05.

The addition of tpMSCs to equine PBMCs did not give rise to a significant increase in mean PBMC proliferation before (PBMCs + tpMSCs; 4.2 ± 2.2%) and after a single tpMSC treatment (PBMCs + tpMSCs; 3.2 ± 1.4%) compared to the negative control (PBMCs; 3.2 ± 1.7% and 3.7 ± 1.5%, respectively). Moreover, no statistically significant difference was present between PBMC proliferation before and after treatment (Figure 1A).

#### Study 2: Repeated tpMSC Treatment

The mean PBMC proliferation percentage of cells from treated horses (PBMCs + tpMSCs; 1.5 ± 0.7%) was significantly lower than the proliferation rate of the negative control samples (PBMCs; 4.2 ± 2.5%) (*P* = 0.041) (Figure 1B).

### Flow Cytometric Crossmatch Assay

#### Study 1: Single tpMSC Treatment

**Figure 2:**
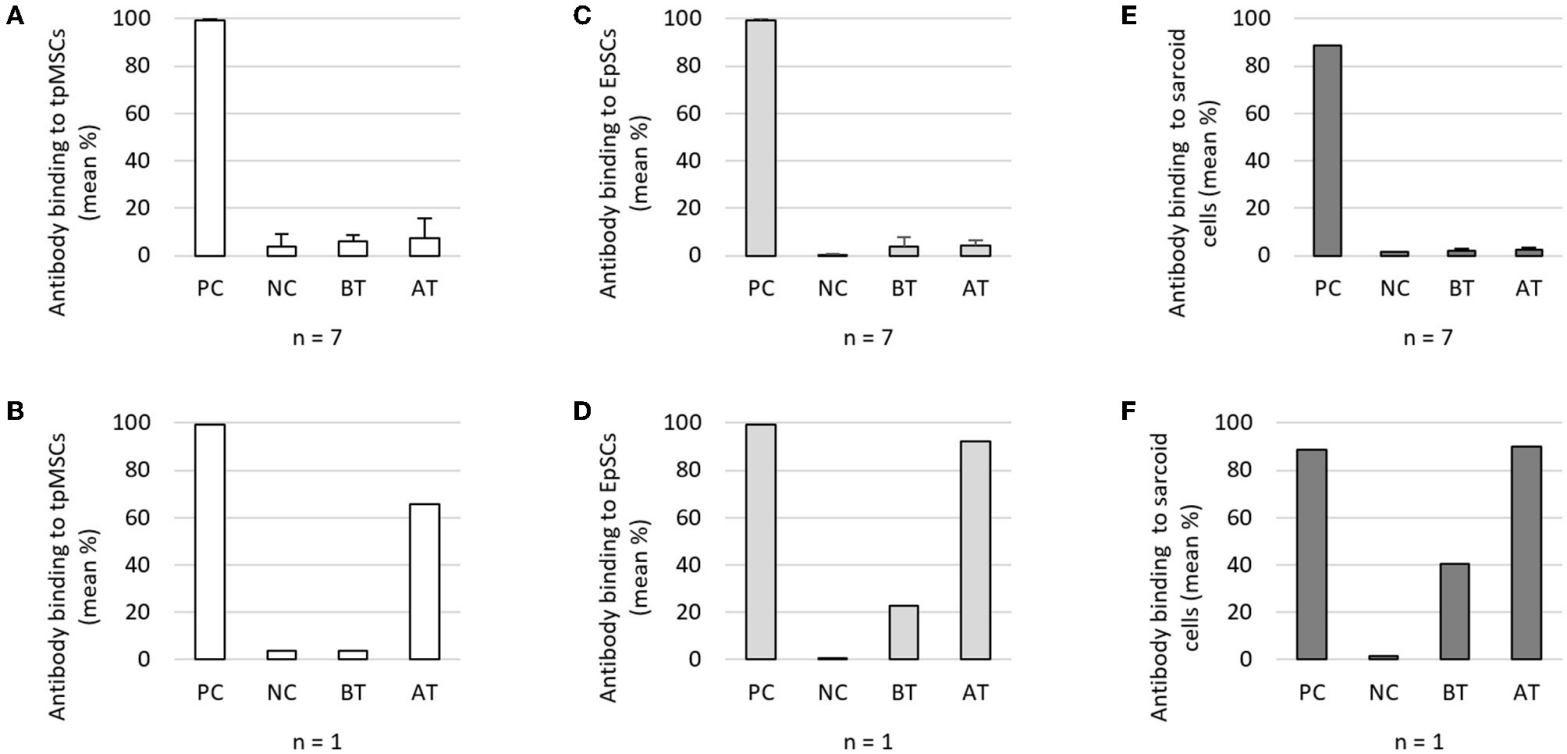
Flow cytometric crossmatch assay study 1. **(A)** Mean (+ SD) antibody binding to tpMSCs in seven out of eight horses and **(B)** one out of eight horses. **(C)** Mean (+ SD) antibody binding to EpSCs in seven out of eight horses and **(D)** one out of eight horses. **(E)** Mean (+ SD) antibody binding to sarcoid cells in seven out of eight horses and **(F)** one out of eight horses. tpMSCs, tenogenic primed mesenchymal stem cells; EpSCs, epithelial-like stem cells; PC, positive control; NC, negative control; BT, before treatment; AT, after treatment.

The percentage antibody binding to tpMSCs obtained before (5.9 ± 2.7%) and after (7.5 ± 8.1%) treatment was similar to the negative control (4.0 ± 4.9%) for seven out of eight horses. For these seven horses, there was no significant difference between the antibody binding to tpMSCs before and after treatment (Figure 2A). One horse showed an elevated level of antibody binding after the treatment with tpMSCs (65.6%) (Figure 2B), which was further investigated. A retrospective investigation of the clinical data of this horse revealed it was diagnosed with an equine sarcoid. To evaluate potential cross-reactivity of the anti-tpMSC antibodies to other cell types an additional FCCA was performed using skin EpSCs and a sarcoid cell line. No significant increase in antibody binding to EpSCs and sarcoid cells was detected after treatment (4.1 ± 2.2% and 2.6 ± 0.8%) compared to before treatment (4.0 ± 3.7% and 2.2 ± 0.7%) in seven out of eight horses (Figures 2C,E). However, a remarkable increase in antibody response against the EpSCs and sarcoid cells after treatment (92.1% and 90.1%) was observed compared to before treatment (22.7% and 40.4%) in the horse with the equine sarcoid (Figures 2D,F).

#### Study 2: Repeated tpMSC Treatment

**Figure 3:**
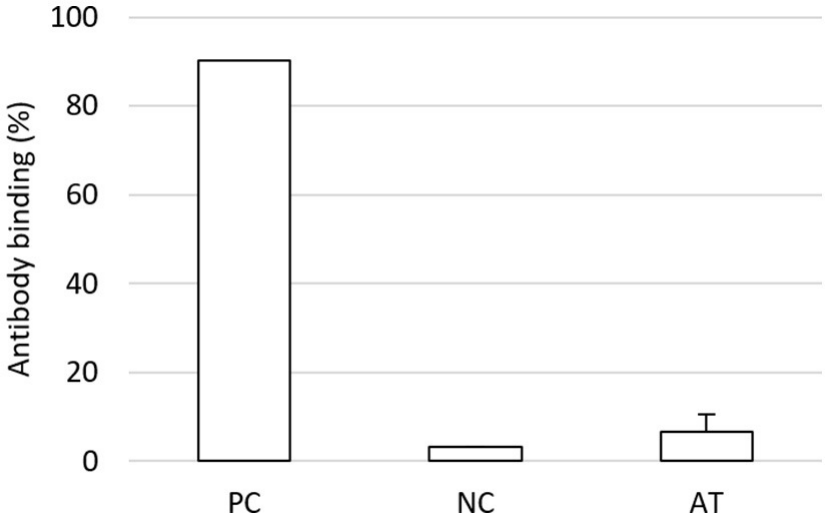
Flow cytometric crossmatch assay study 2. In six horses suffering from naturally occurring tendon injuries treated two times with tpMSCs, no alloantibodies against the tpMSCs could be detected in the equine serum. tpMSCs, tenogenic primed mesenchymal stem cells; PC, positive control; NC, negative control; AT, after treatment.

In the repeatedly treated horses, no alloantibodies against the tpMSCs could be detected in the equine serum after the second treatment (6.7 ± 3.7%) (Figure 3). From these horses, no baseline samples before treatment could be obtained for comparison.

### Anti-BSA Antibody ELISA

**Figure 4:**
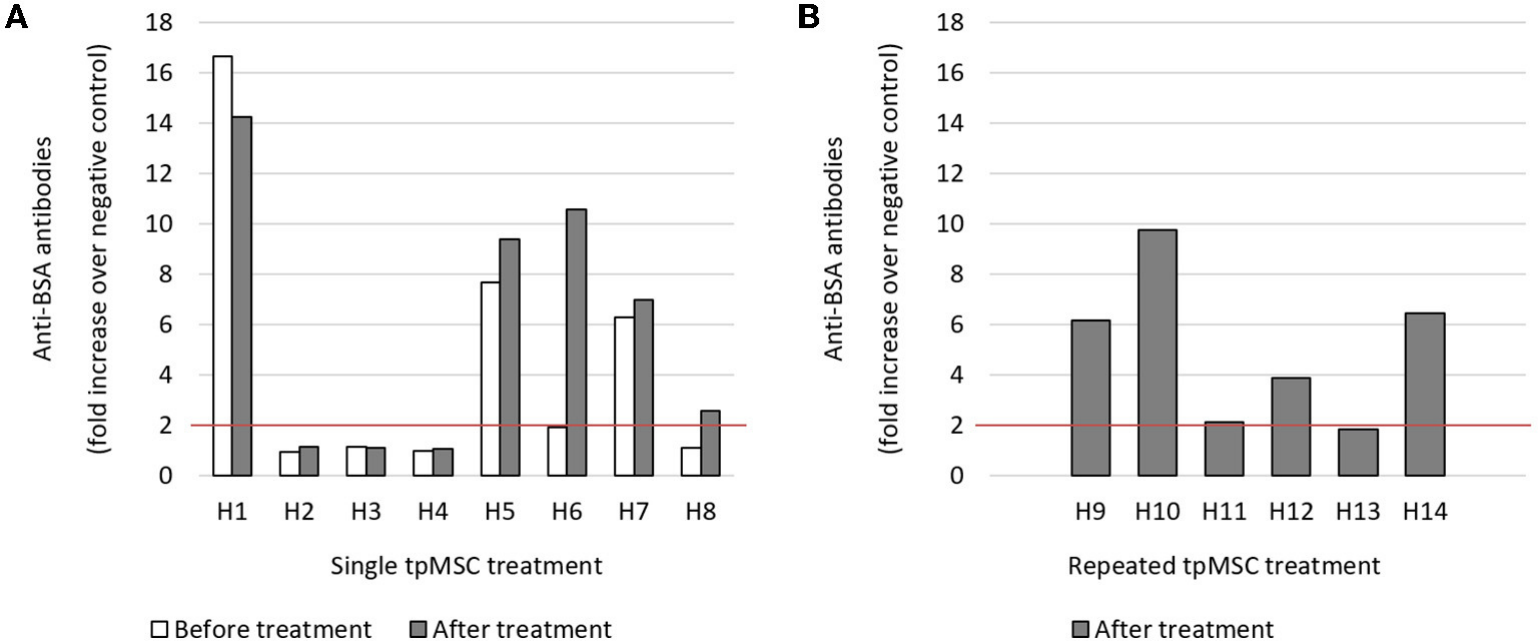
Anti-BSA antibody detection. **(A)** In three out of eight horses before and after a single tpMSC treatment, a high preexisting anti-BSA antibody level could be detected. One horse showed a 5 times higher antibody level after treatment (H6). **(B)** In four out of six horses after two local tpMSC treatments a high anti-BSA antibody level could be detected. tpMSC, tenogenic primed mesenchymal stem cell.

In study 1, three out of eight horses had high preexisting anti-BSA antibody levels ranging from 6 to 17 times higher than the horses with no anti-BSA antibody titers before tpMSC administration, which is defined as a fold increase < 2 compared to the negative control (15). Four of the five horses did not show considerable changes in anti-BSA antibody levels when sampled before or after tpMSC administration. However, one of these five horses showed a 5 times higher antibody level after treatment (Figure 4A, H6).

In the horses that received a repeated tpMSC injection (study 2), four out of six had high anti-BSA antibody titers after two treatments and one horse had a slightly increased anti-BSA antibody titer (Figure 4B, H11).

### Bovine Papillomavirus PCR

A PCR was performed on the sarcoid cell line, EpSC line and MSCs derived from the tpMSC donor to evaluate for presence of BPV-1 and BPV-2 DNA. A positive result was only obtained in the equine sarcoid cells (949,400 copies/μL), indicating that BPV was not the common denominator and the equine sarcoid cells were indeed a clinically relevant cell line (Table 2). All cell types were negative for BPV-2.

**Table 2:** PCR results of the BPV detection in EpSCs, MSCs and equine sarcoid cells.

**Gene**	**Sample**	**Cq value**	**Starting quantity (copies/μL)**	**Log starting quantity (copies/μL)**
IFN-β(house-keeping gene)	EpSCs	20.6	81,200	4.9
	MSCs	19.2	194,050	5.3
	Sarcoid cells	19.8	126,700	5.1
BPV-1	EpSCs	Not applicable	Not applicable	Not applicable
	MSCs	Not applicable	Not applicable	Not applicable
	Sarcoid cells	19.8	949,400	6.0
BPV-2	EpSCs	Not applicable	Not applicable	Not applicable
	MSCs	Not applicable	Not applicable	Not applicable
	Sarcoid cells	Not applicable	Not applicable	Not applicable

## Discussion

To the best of our knowledge, this is the first study in the equine species investigating cellular and humoral immunogenicity of single or repeated intralesional injections of equine allogeneic tenogenic primed peripheral blood-derived MSCs (tpMSCs) in horses suffering from tendon injuries. The absence of the cellular immune response was evaluated in a modified MLR assay resulting in a similar mean recipient PBMC proliferation when compared to the negative control. These results clearly indicate that allogeneic tpMSCs can be injected intralesional without the induction of a cellular immune response. Previously published MLR results of allogeneic bone marrow-derived MSCs (BM-MSCs) co-cultured with equine PBMCs also described absence of the host's cellular immune response (13, 37). In a comparable *in vitro* study, the cellular immunogenicity of autologous and allogeneic BM-MSCs was investigated (39). It was observed that the allogeneic MSCs did not generate a strong alloreactive T cell response. Additionally, an *in vitro* study reported by Ranera et al. shows that equine allogeneic BM-MSCs are able to suppress the proliferation of mismatched PBMCs (16). However, this was not the case with MHC-II positive BM-MSCs which elicited a significantly increased responder lymphocyte proliferation (32), indicating that immunogenicity investigations remain important when evaluating MSC treatment options (38, 39). Considering humoral immunogenicity, one out of eight horses which received a single local allogenic tpMSC treatment and none of the horses receiving repeated treatments had anti-tpMSC antibodies. This was observed by the absence of a significant difference between the antibody binding before and after treatment (Study 1) or between the negative control and after treatment (Study 2). One horse (Study 1; H6; 12,5%) had an elevated antibody binding to the tpMSCs after a single treatment and was found to also have an equine sarcoid [i.e. the most common dermatological neoplasm in horses (52)]. The results of the additional analyses revealed that the humoral immune response induced by tpMSC treatment showed cross-reaction with EpSCs, sarcoid cells and bovine xenoproteins (BSA). Furthermore, a pre-existing presence of antibodies against sarcoid cells and other cell types of the epidermal lineage (i.e. EpSCs) were observed in this single horse. Serum from the other seven horses revealed no such binding or changes in anti-BSA antibody levels before and after tpMSC treatment. If there are antibodies present against BSA, this should always be taken under consideration when treating patients with a cell-based therapy cultivated in culture media supplemented with BSA. If cross-reaction of these specific antibodies occurs with the applied cell therapy, this could lead to a humoral immune response against the treatment.

In the current study, different timepoints were used for the evaluation of the cellular and humoral immune response. For the humoral immune response, it has been reported that the highest antibody peak after a single treatment is expected two weeks after treatment (53). Therefore, blood was sampled two weeks after the single treatment in the first study for the humoral immune response. When administering multiple injections, it has been reported that the antibody peak remains high from one week after the second treatment administration onwards (53). Therefore, blood was drawn six to eight weeks after the last treatment of the repeatedly treated horses from the second study. Considering the cellular immune response, lymphocyte responses and memory cells are shown to persist on a long-term (54, 55). Therefore, the blood collection at the end of the first study could also be used to perform the mixed lymphocyte reaction assay. For the repeated injected horses, the blood collection performed at week 6–8 to investigate the humoral immune response was also used to perform the mixed lymphocyte reaction.

Additionally, BPV is a well-known pathogen associated with the development of equine sarcoids (11). As a result of the PCR analysis for the detection of BPV DNA in MSCs, EpSCs and a sarcoid cell line, a positive result was only obtained in the sarcoid line, indicating BPV was not the common denominator and the sarcoid cell line was indeed a relevant cell line of an in life sarcoid. Based on current results, it remains unclear whether this could be related to the presence of a naturally occurring equine sarcoid in one sero-converting horse or if it is an individual non-specific immune response against one or several different (xeno)antigens. Despite the occurrence of the non-specific immune response in one horse, a previous study performed by our group including these horses revealed no clinical abnormalities and promising results of the tpMSC treatment (46). Owens et al., reported an anti-BM-MSC antibody response in 37% of the horses receiving an allogeneic treatment (15). In the latter *in vivo* study, a total of 19 horses received fully unmatched allogeneic adipose tissue-derived MSCs or BM-MSCs via intravenous, intra-arterial, intra-tendon and intraocular routes (range of 25 to 80 million MSCs per injection). The same flow cytometric crossmatch assay was used to detect alloantibodies. The high MSC dosage in this study may contribute to the higher alloantibody response when compared to the current investigation. Additionally, Barrachina et al., reported alloantibody production in case of a MHC halfmatch and mismatch in the sera of recipient horses following a single and repeated intra-articular allogeneic BM-MSC administration using an *in vitro* microcytoxicity assay (34). In the latter study, the horses received multiple BM-MSC injections with only 1 week, 3 weeks or 90 days between treatments. This in combination with the use of a different detection method could give rise to a higher antibody detection.

Furthermore, in clinical studies, the allogeneic use of MSCs has been described for single (12, 23, 46) and repeated (24, 25) treatment of horses suffering from tendon injuries without evidence of any local immunological reactions (absence of heat, swelling, pain or lameness) after intralesional injection. On the other hand, a higher number of circulating CD8 T-cells after repeated intravenous injection of equine allogeneic MSCs has been reported (56, 57). The authors suggested this could be an alloantigen-directed cytotoxic response. Although it has been described that repeated administration of allogeneic peripheral blood-derived tpMSCs is indicated in some clinical cases to improve the outcome of tendon injuries (24, 25), in depth research on the cellular and humoral immunogenicity of repeated allogeneic MSC treatments is presently lacking, emphasizing the importance of current research.

The performed study comes with some limitations. First, from the horses with naturally occurring tendon lesions only serum after the second tpMSC injection and no serum before the first or second tpMSC injection was available. Therefore, baseline values could not be included for these horses. However, these time points were investigated in the horses receiving a single tpMSC treatment. Additionally, since these horses are client-owned and not involved in a clinical trial with prescheduled visits, no standardized follow-up time points were included as in Study 1. Second, considering the lack of positive results in the MLR assay, the use of PBMCs derived from the allogeneic donor could be included as a positive control and autologous PBMCs as a negative control to support the results. This way the PBMC proliferation induced by allogeneic cells can be evaluated as positive control in addition to the concanavalin A stimulation. However, a previous study performed by our group showed that native MSCs are able to suppress concanavalin A stimulated PBMCs (58). This data shows that if there is a reaction between the MSCs and PBMCs in the co-culture, this can be detected using this assay. Third, both studies consisted of respectively 8 or 6 horses, which is a rather small sample size. Therefore, these results should be confirmed in a larger field study. Fourth, the equine leukocyte antigen (ELA) haplotype from both donor and recipient horses was unknown. It has been reported that specific antibodies can be produced when donor and recipient are ELA-mismatched, but not when they are ELA-matched (33, 34, 40). It is thus possible that anti-ELA antibodies are produced when donor and recipient are ELA-mismatched. However regarding the clinical applicability of MSCs it is not always possible in the field to only treat ELA-matched recipients when an MSC treatment is indicated.

It can be concluded that, except for one horse, the administration of tpMSCs did not induce a cellular or humoral immune response following an intralesional single or repeated (two consecutive) allogeneic tpMSC treatment in horses with tendon injury, confirming the low immunogenic properties of equine allogeneic peripheral blood-derived mesenchymal stem cells in these two studies. In the future, a larger field study should confirm these findings and support the safe use of equine allogeneic tpMSCs as a promising therapeutic for horses suffering from naturally occurring tendon injuries.

## Data Availability Statement

The original contributions presented in the study are included in the article/Supplementary Material, further inquiries can be directed to the corresponding author/s.

## Ethics Statement

The animal study was reviewed and approved by Independent Ethics Committee approved by the Flemish Government (permit number: LA1700607). Written informed consent was obtained from the owners for the participation of their animals in this study.

## Author Contributions

JSp, LV, and ED conceived the study and planned the design. Mixed lymphocyte reaction and alloantibody assays were performed by ED and LV. The anti-BSA antibody assays were performed by MP and LDa. Statistical analysis was done by LDu and LV. ED wrote the first draft of the manuscript. JSp, SB, JSa, LV, AM, FP, and KC provided supervision and critical review of the manuscript. All authors read and approved the final manuscript.

## Funding

This research was funded both by BIVMB supported by a grant from the Flanders Innovation & Entrepreneurship (Vlaio project number: HBC.2018.0182).

## Conflict of Interest

JHS declares competing financial interests as shareholder in Boehringer Ingelheim Veterinary Medicine Belgium (BIVMB) at the time of the study. SB, JHS, ED, and LV were all employed by BIVMB at the time of the study. The content of this manuscript contains a stem cell product under development (RenuTend^®^) owned and patented by BIVMB. The remaining authors declare that the research was conducted in the absence of any commercial or financial relationships that could be construed as a potential conflict of interest.

## Publisher's Note

All claims expressed in this article are solely those of the authors and do not necessarily represent those of their affiliated organizations, or those of the publisher, the editors and the reviewers. Any product that may be evaluated in this article, or claim that may be made by its manufacturer, is not guaranteed or endorsed by the publisher.

## Abbreviations

7-AAD: 7-aminoactinomycine D
BM: Bone marrow
BPV: Bovine papillomavirus
BSA: Bovine serum albumin
DMEM: Dulbecco's modified Eagle medium
EpSC: Epithelial-like stem cell
FBS: Fetal bovine serum
FCCA: Flow cytometric crossmatch assay
HBSS: Hank's balanced salt solution
MHC: Major histocompatibility complex
MLR: Mixed lymphocyte reaction
MSC(s): Mesenchymal stem cell(s)
PBMC(s): Peripheral blood mononuclear cell(s)
SDFT: Superficial digital flexor tendon
tpMSC(s): Tenogenic primed mesenchymal stem cell(s).

## References

[1] Smith RKW Werling NJ Dakin SG Alam R Goodship AE Dudhia J. Beneficial effects of autologous bone marrow-derived mesenchymal stem cells in naturally occurring tendinopathy. PLoS ONE. (2013) 8:1–14. 10.1371/annotation/a30a4b87-8904-4510-b0a8-5b6ca6097f9a 24086616 PMC3783421

[2] Conze P Van Schie HT Weeren RV Staszyk C Conrad S Skutella T. Effect of autologous adipose tissue-derived mesenchymal stem cells on neovascularization of artificial equine tendon lesions. Regen Med. (2014) 9:743–57. 10.2217/rme.14.55 25431911

[3] Romero A Barrachina L Ranera B Remacha AR Moreno B de Blas I. Comparison of autologous bone marrow and adipose tissue derived mesenchymal stem cells, and platelet rich plasma, for treating surgically induced lesions of the equine superficial digital flexor tendon. Vet J. (2017) 224:76–84. 10.1016/j.tvjl.2017.04.005 28697880

[4] Bertoni L Branly T Jacquet S Desancé M Desquilbet L Rivory P. Intra-articular injection of 2 different dosages of autologous and allogeneic bone marrow- and umbilical cord-derived mesenchymal stem cells triggers a variable inflammatory response of the fetlock joint on 12 sound experimental horses. Stem Cells Int. (2019) 9431894. 10.1155/2019/9431894 31191689 PMC6525957

[5] Shokry M Mostafo A Tohamy A El-Sharkawi M. Autologous mesenchymal stem cells for treatment of acute superficial digital flexor tendonitis in athletic horses- a clinical study of 1 5 cases. Pferdeheilkunde. (2020) 36:43–8. 10.21836/PEM20200107

[6] Murphy JM Dixon K Beck S Fabian D Feldman A Barry F. Reduced chondrogenic and adipogenic activity of mesenchymal stem cells from patients with advanced osteoarthritis. Arthritis Rheum. (2002) 46:704–13. 10.1002/art.10118 11920406

[7] Nie Y Lau CS Lie AK Chan GC Mok MY. Defective phenotype of mesenchymal stem cells in patients with systemic lupus erythematosus. Lupus. (2010) 19:850–859. 10.1177/0961203309361482 20511276

[8] Choudhery MS Khan M Mahmood R Mehmood A Khan SN Riazuddin S. Bone marrow derived mesenchymal stem cells from aged mice have reduced wound healing, angiogenesis, proliferation and anti-apoptosis capabilities. Cell Biol Int. (2012) 36:747–53. 10.1042/CBI20110183 22352320

[9] Marycz K Kornicka K Basinska K Czyrek A. Equine metabolic syndrome affects viability, senescence, and stress factors of equine adipose-derived mesenchymal stromal stem cells: new insight into EqASCS isolated from EMS horses in the context of their aging. Oxid Med Cell Longev. (2016) 2016:4710326. 10.1155/2016/4710326 26682006 PMC4670679

[10] Marycz K Kornicka K Maredziak M Golonka P Nicpoń J. Equine metabolic syndrome impairs adipose stem cells osteogenic differentiation by predominance of autophagy over selective mitophagy. J Cell Mol Med. (2016) 20:2384–404. 10.1111/jcmm.12932 27629697 PMC5134411

[11] Chambers G Ellsmore VA O'Brien PM Reid SWJ Love S Campo MS. Association of bovine papillomavirus with the equine sarcoid. J Gen Virol. (2003) 84:1055–62. 10.1099/vir.0.18947-0 12692268

[12] Ricco S Renzi S Del Bue M Conti V Merli E Ramoni R. Allogeneic adipose tissue-derived mesenchymal stem cells in combination with platelet rich plasma are safe and effective in the therapy of superficial digital flexor tendonitis in the horse. Int J Immunopathol Pharmacol. (2013) 26:61–8. 10.1177/03946320130260S108 24046950

[13] Paterson YZ Rash N Garvican ER Paillot R Guest DJ. Equine mesenchymal stromal cells and embryo-derived stem cells are immune privileged *in vitro*. Stem Cell Res Ther. (2014) 5. 10.1186/scrt479 25080326 PMC4247727

[14] Van Loon VJF Scheffer CJW Genn HJ Hoogendoorn AC Greve JW. Clinical follow-up of horses treated with allogeneic equine mesenchymal stem cells derived from umbilical cord blood for different tendon and ligament disorders. Vet Q. (2014) 34:92–7. 10.1080/01652176.2014.949390 25072527

[15] Owens SD Kol A Walker NJ Borjesson DL. Allogeneic Mesenchymal Stem Cell Treatment Induces Specific Alloantibodies in Horses. Stem Cells Int. (2016) 5830103. 10.1155/2016/5830103 27648075 PMC5018342

[16] Ranera B Antczak D Miller D Doroshenkova T Ryan A Mcilwraith CW. Donor-derived equine mesenchymal stem cells suppress proliferation of mismatched lymphocytes. Equine Vet J. (2016) 48:253–60. 10.1111/evj.12414 25582202

[17] Brandt L Schubert S Scheibe P Brehm W Franzen J Gross C. Tenogenic properties of mesenchymal progenitor cells are compromised in an inflammatory environment. Int J Mol Sci. (2018) 19:1–26. 10.3390/ijms19092549 30154348 PMC6163784

[18] Harris MT Butler DL Boivin GP Florer JB Schantz EJ Wenstrup RJ. Mesenchymal stem cells used for rabbit tendon repair can form ectopic bone and express alkaline phosphatase activity in constructs. J Orthop Res. (2004) 22:998–1003. 10.1016/j.orthres.2004.02.012 15304271

[19] Dressler MR Butler DL Boivin GP. Effects of age on the repair ability of mesenchymal stem cells in rabbit tendon. J Orthop Res. (2005) 23:287–93. 10.1016/j.orthres.2004.06.017 15734238

[20] Fang Z Zhu T Shen WL Tang QM Chen JL Yin Z. Transplantation of fetal instead of adult fibroblasts reduces the probability of ectopic ossification during tendon repair. Tissue Eng - Part A. (2014) 20:1815–26. 10.1089/ten.tea.2013.0296 24410299 PMC4086799

[21] Shojaee A Parham A. Strategies of tenogenic differentiation of equine stem cells for tendon repair: Current status and challenges. Stem Cell Res Ther. (2019) 10:1–13. 10.1186/s13287-019-1291-0 31215490 PMC6582602

[22] Gomiero C Bertolutti G Martinello T Van Bruaene N Broeckx SY Patruno M. Tenogenic induction of equine mesenchymal stem cells by means of growth factors and low-level laser technology. Vet Res Commun. (2016) 40:39–48. 10.1007/s11259-016-9652-y 26757735

[23] Broeckx S Zimmerman M Aerts D Seys B Suls M Mariën T. Tenogenesis of equine peripheral blood-derived mesenchymal stem cells: *In vitro* versus *In vivo*. J Tissue Sci Eng S11. (2012) 1–6. 10.4172/2157-7552.S11-001

[24] Vandenberghe A Broeckx SY Beerts C Seys B Zimmerman M Verweire I. Tenogenically induced allogeneic mesenchymal stem cells for the treatment of proximal suspensory ligament desmitis in a horse. Front Vet Sci. (2015) 2. 10.3389/fvets.2015.00049 26664976 PMC4672201

[25] Beerts C Suls M Broeckx SY Seys B Vandenberghe A Declercq J. Tenogenically induced allogeneic peripheral blood mesenchymal stem cells in allogeneic platelet-rich plasma: 2-year follow-up after tendon or ligament treatment in horses. Front Vet Sci. (2017) 4:1–10. 10.3389/fvets.2017.00158 29018808 PMC5622984

[26] Broeckx S Suls M Beerts C Vandenberghe A Seys B Wuertz-Kozak K. Allogenic mesenchymal stem cells as a treatment for equine degenerative joint disease: a pilot study. Curr Stem Cell Res Ther. (2014) 9:497–503. 10.2174/1574888X09666140826110601 25175766

[27] Broeckx SY Spaas JH Chiers K Duchateau L Van Hecke L Van Brantegem L. Equine allogeneic chondrogenic induced mesenchymal stem cells: a GCP target animal safety and biodistribution study. Res Vet Sci. (2018) 117:246–54. 10.1016/j.rvsc.2017.12.018 29329028

[28] Broeckx SY Martens AM Bertone AL Van Brantegem L Duchateau L Van Hecke L. The use of equine chondrogenic-induced mesenchymal stem cells as a treatment for osteoarthritis: A randomised, double-blinded, placebo-controlled proof-of-concept study. Equine Vet J. (2019) 51:787–94. 10.1111/evj.13089 30815897 PMC6850029

[29] Broeckx S Zimmerman M Crocetti S Suls M Mariën T Ferguson SJ. Regenerative therapies for equine degenerative joint disease: a preliminary study. PLoS ONE. (2014) 9:1–11. 10.1371/journal.pone.0085917 24465787 PMC3896436

[30] Ardanaz N Vázquez FJ Romero A Remacha AR Barrachina L Sanz A. Inflammatory response to the administration of mesenchymal stem cells in an equine experimental model: Effect of autologous, and single and repeat doses of pooled allogeneic cells in healthy joints. BMC Vet Res. (2016) 12:1–9. 10.1186/s12917-016-0692-x 27029614 PMC4815220

[31] Joswig AJ Mitchell A Cummings KJ Levine GJ Gregory CA Smith R. Repeated intra-articular injection of allogeneic mesenchymal stem cells causes an adverse response compared to autologous cells in the equine model. Stem Cell Res Ther. (2017) 8:42. 10.1186/s13287-017-0503-8 28241885 PMC5329965

[32] Schnabel LV Pezzanite LM Antczak DF Felippe MJB Fortier LA. Equine bone marrow-derived mesenchymal stromal cells are heterogeneous in MHC class II expression and capable of inciting an immune response in vitro. Stem Cell Res Ther. (2014) 5:1–13. 10.1186/scrt402 24461709 PMC4055004

[33] Pezzanite LM Fortier LA Antczak DF Cassano JM Brosnahan MM Miller D. Equine allogeneic bone marrow-derived mesenchymal stromal cells elicit antibody responses *in vivo*. Stem Cell Res Ther. (2015) 6:1–11. 10.1186/s13287-015-0053-x 25889095 PMC4414005

[34] Barrachina L Cequier A Romero A Vitoria A Zaragoza P Vázquez FJ. Allo-antibody production after intraarticular administration of mesenchymal stem cells (MSCs) in an equine osteoarthritis model: Effect of repeated administration, MSC inflammatory stimulation, and equine leukocyte antigen (ELA) compatibility. Stem Cell Res Ther. (2020) 11:1–12. 10.1186/s13287-020-1571-8 32028995 PMC7006079

[35] Noronha NDC Mizukami A Caliári-Oliveira C Cominal JG Rocha JLM Covas DT. Correction to: Priming approaches to improve the efficacy of mesenchymal stromal cell-based therapies. Stem Cell Res Ther. (2019) 10:1–21. 10.1186/s13287-019-1259-0 31101067 PMC6525348

[36] Iyer SS Rojas M. Anti-inflammatory effects of mesenchymal stem cells: novel concept for future therapies. Expert Opin Biol Ther. (2008) 8:569–81. 10.1517/14712598.8.5.569 18407762

[37] Pigott JH Ishihara A Wellman ML Russell DS Bertone AL. Investigation of the immune response to autologous, allogeneic, and xenogeneic mesenchymal stem cells after intra-articular injection in horses. Vet Immunol Immunopathol. (2013) 156:99–106. 10.1016/j.vetimm.2013.09.003 24094688

[38] Carrade DD Affolter VK Outerbridge CA Watson JL Galuppo LD Buerchler S. Intradermal injections of equine allogeneic umbilical cord-derived mesenchymal stem cells are well tolerated and do not elicit immediate or delayed hypersensitivity reactions. Cytotherapy. (2011) 13:1180–92. 10.3109/14653249.2011.602338 21899391

[39] Colbath AC Dow SW Phillips JN McIlwraith CW Goodrich LR. Autologous and allogeneic equine mesenchymal stem cells exhibit equivalent immunomodulatory properties *in vitro*. Stem Cells Dev. (2017) 26:503–11. 10.1089/scd.2016.0266 27958776

[40] Berglund AK Schnabel LV. Allogeneic major histocompatibility complex-mismatched equine bone marrow-derived mesenchymal stem cells are targeted for death by cytotoxic anti-major histocompatibility complex antibodies. Equine Vet J. (2017) 49:539–44. 10.1111/evj.12647 27862236 PMC5425313

[41] Barrachina L Remacha AR Romero A Vitoria A Albareda J Prades M. Assessment of effectiveness and safety of repeat administration of proinflammatory primed allogeneic mesenchymal stem cells in an equine model of chemically induced osteoarthritis. BMC Vet Res. (2018) 14:1–17. 10.1186/s12917-018-1556-3 30119668 PMC6098603

[42] Cabon Q Febre M Gomez N Cachon T Pillard P Carozzo C. Long-term safety and efficacy of single or repeated intra-articular injection of allogeneic neonatal mesenchymal stromal cells for managing pain and lameness in moderate to severe canine osteoarthritis without anti-inflammatory pharmacological support: Pi. Front Vet Sci. (2019) 6:1–14. 10.3389/fvets.2019.00010 30805348 PMC6371748

[43] Magri C Schramme M Febre M Cauvin E Labadie F Saulnier N. Comparison of efficacy and safety of single versus repeated intra-articular injection of allogeneic neonatal mesenchymal stem cells for treatment of osteoarthritis of the metacarpophalangeal/metatarsophalangeal joint in horses: a clinical pilot study. PLoS ONE. (2019) 14:1–16. 10.1371/journal.pone.0221317 31465445 PMC6715221

[44] Paillot R Marcillaud Pitel C D'ablon X Pronost S. Equine vaccines: how, when and why? Report of the vaccinology session, french equine veterinarians association, 2016, reims. Vaccines. (2017) 5:46. 10.3390/vaccines5040046 29207516 PMC5748612

[45] Gershwin LJ Netherwood KA Norris MS Behrens NE Shao MX. Equine IgE responses to non-viral vaccine components. Vaccine. (2012) 30:7615–20. 10.1016/j.vaccine.2012.10.029 23088888

[46] Depuydt E Broeckx SY Van Hecke L Chiers K Van Brantegem L van Schie H. The evaluation of equine allogeneic tenogenic primed mesenchymal stem cells in a surgically induced superficial digital flexor tendon lesion model. Front Vet Sci. (2021) 8:166. 10.3389/fvets.2021.641441 33748217 PMC7973085

[47] Spaas JH De Schauwer C Cornillie P Meyer E Van Soom A Van de Walle GR. Culture and characterisation of equine peripheral blood mesenchymal stromal cells. Vet J. (2013) 195:107–13. 10.1016/j.tvjl.2012.05.006 22717781

[48] Muul LM Heine G Silvin C James SP Candotti F Radbruch A. Measurement of proliferative responses of cultured lymphocytes. Curr Protoc Immunol. (2011) 94:7.10.1–26. 10.1002/0471142735.im0710s94 18729064

[49] Van Hecke L Magri C Duchateau L Beerts C Geburek F Suls M. Repeated intra-articular administration of equine allogeneic peripheral blood-derived mesenchymal stem cells does not induce a cellular and humoral immune response in horses. Vet Immunol Immunopathol. (2021) 239:110306. 10.1016/j.vetimm.2021.110306 34365135

[50] Broeckx SY Maes S Martinello T Aerts D Chiers K Mariën T. Equine epidermis: A source of epithelial-like stem/progenitor cells with *in vitro* and *in vivo* regenerative capacities. Stem Cells Dev. (2014) 23:1134–48. 10.1089/scd.2013.0203 24368059

[51] Dominici M Le Blanc K Mueller I Slaper-Cortenbach I Marini FC Krause DS. Minimal criteria for defining multipotent mesenchymal stromal cells. Int. Soc. Cell Therapy. (2006) 8:315–7. 10.1080/14653240600855905 16923606

[52] Bogaert L Martens A Depoorter P Gasthuys F. Equine sarcoids - Part 1: clinical presentation and epidemiology. Vlaams Diergeneeskd Tijdschr. (2008) 77:2–9.

[53] Murphy KM. Dynamics of adaptive immunity. in Janeway's Immunobiology (Garland Science). (2012) p. 429–464.

[54] Ullenhag GJ Frödin JE Mosolits S Kiaii S Hassan M Bonnet MC. Immunization of colorectal carcinoma patients with a recombinant canarypox virus expressing the tumor antigen Ep-CAM/KSA (ALVAC-KSA) and granulocyte macrophage colony-stimulating factor induced a tumor-specific cellular immune response. Clin Cancer Res. (2003) 9:2447–56. 12855617

[55] Zia A Singh D Saxena S Umrao J Baluni M Ghildiyal S. Detection of long term cellular immune response to Japanese encephalitis vaccination using IFN-γ ELIspot assay. J Med Virol. (2017) 89:2235–8. 10.1002/jmv.24893 28671301

[56] Kol A Wood JA Carrade Holt DD Gillette JA Bohannon-Worsley LK Puchalski SM. Multiple intravenous injections of allogeneic equine mesenchymal stem cells do not induce a systemic inflammatory response but do alter lymphocyte subsets in healthy horses. Stem Cell Res Ther. (2015) 6:1–9. 10.1186/s13287-015-0050-0 25888916 PMC4446064

[57] Kamm JL Riley CB Parlane N Gee EK McIlwraith CW. Interactions between allogeneic mesenchymal stromal cells and the recipient immune system: a comparative review with relevance to equine outcomes. Front Vet Sci. (2021) 7:1–10. 10.3389/fvets.2020.617647 33521090 PMC7838369

[58] Debosschere Y Depuydt E Pauwelyn G Beerts C Van Hecke L Verhaert L. Safety and immunomodulatory properties of equine peripheral blood-derived mesenchymal stem cells in healthy cats. Vet Immunol Immunopathol. (2020) 227:110083. 10.1016/j.vetimm.2020.110083 32563854

