# Supplementary figures and images for: Cellular and Humoral Immunogenicity Investigation of Single and Repeated Allogeneic Tenogenic Primed Mesenchymal Stem Cell Treatments in Horses Suffering From Tendon Injuries
Source: Front Vet Sci. 2022 Feb 24;8:789293. doi: 10.3389/fvets.2021.789293 (PMC8907452; doi:10.3389/fvets.2021.789293)

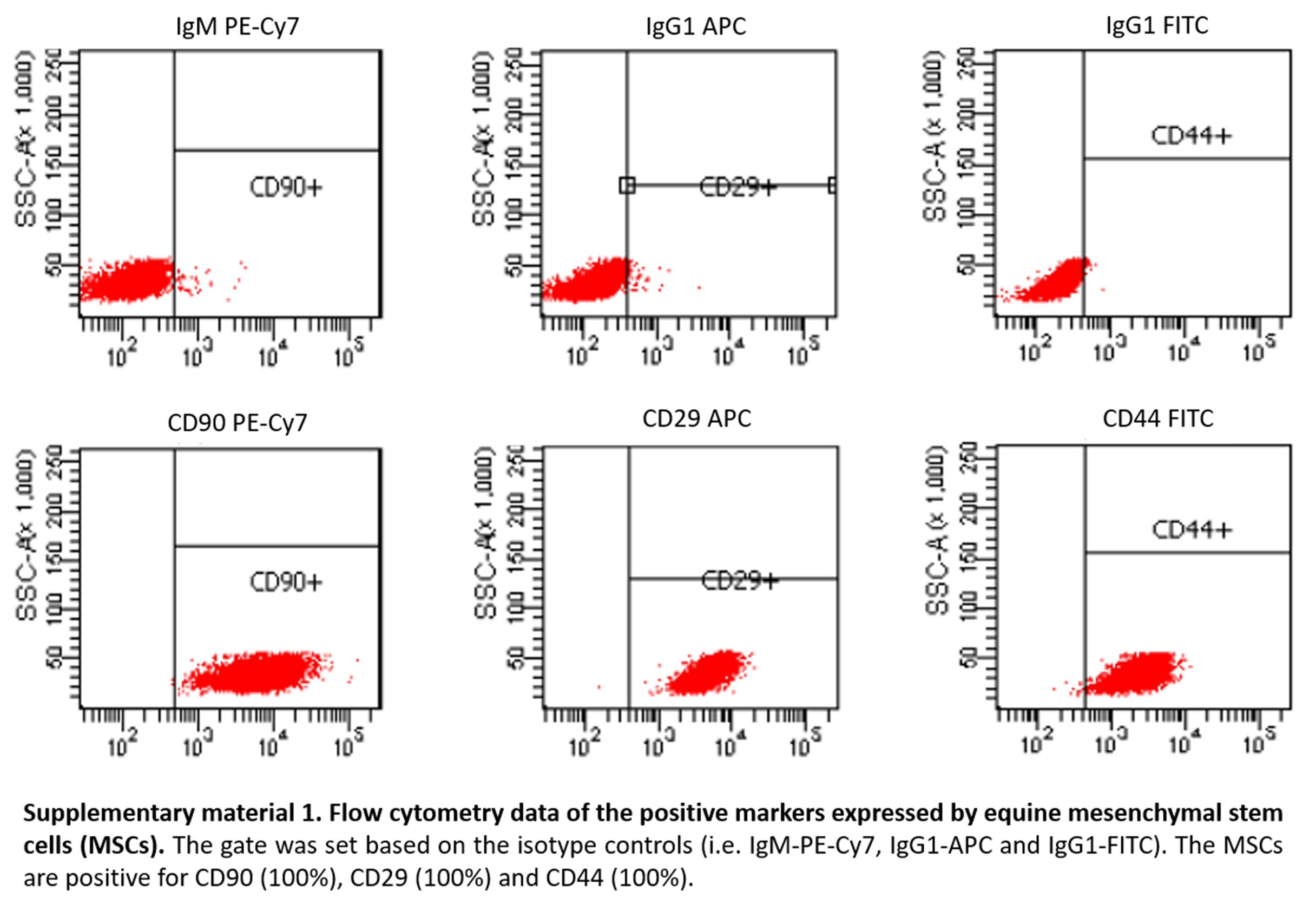

Supplement: Supplementary file 1 [file Image_1.PNG]

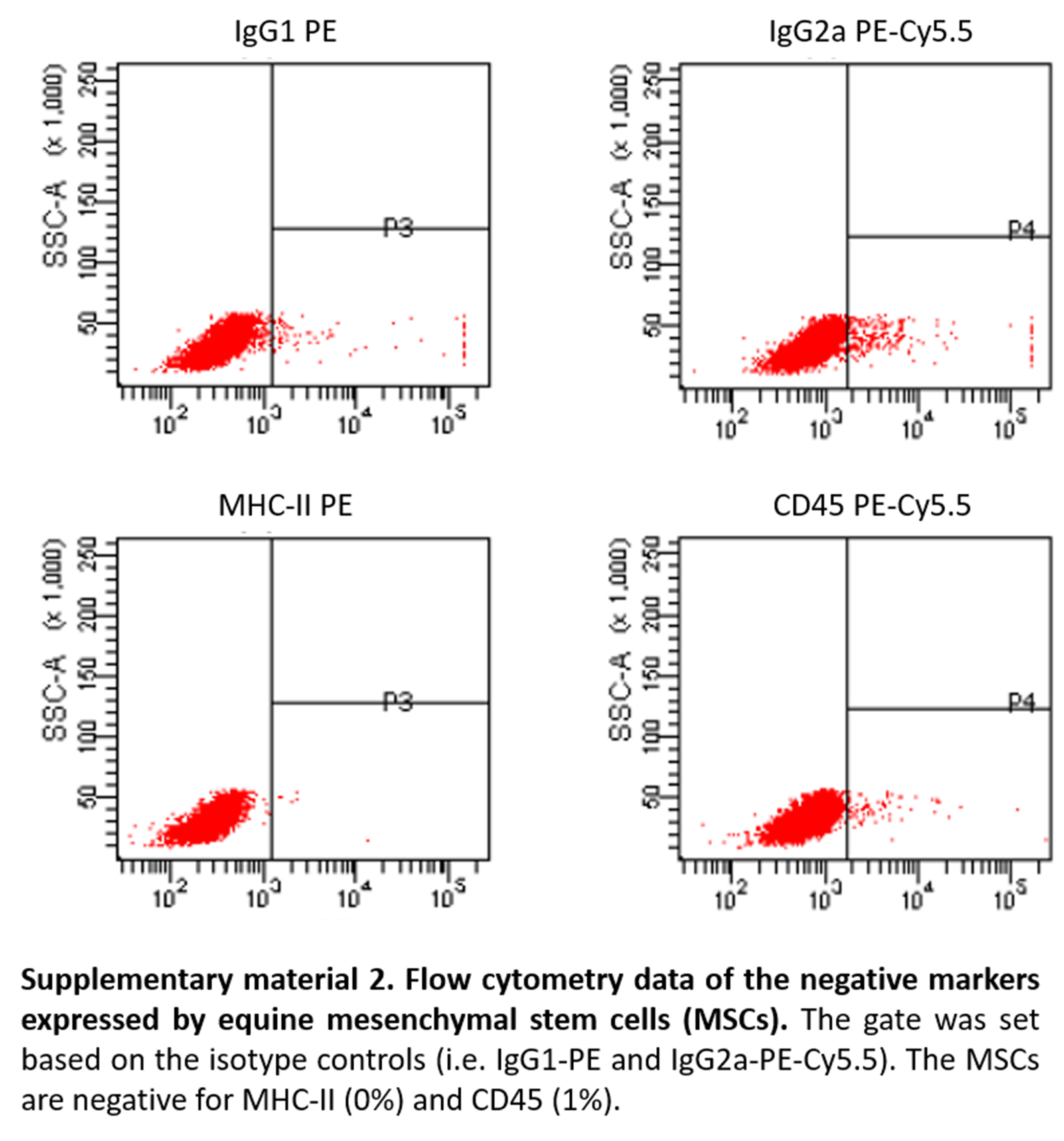

Supplement: Supplementary file 2 [file Image_2.PNG]

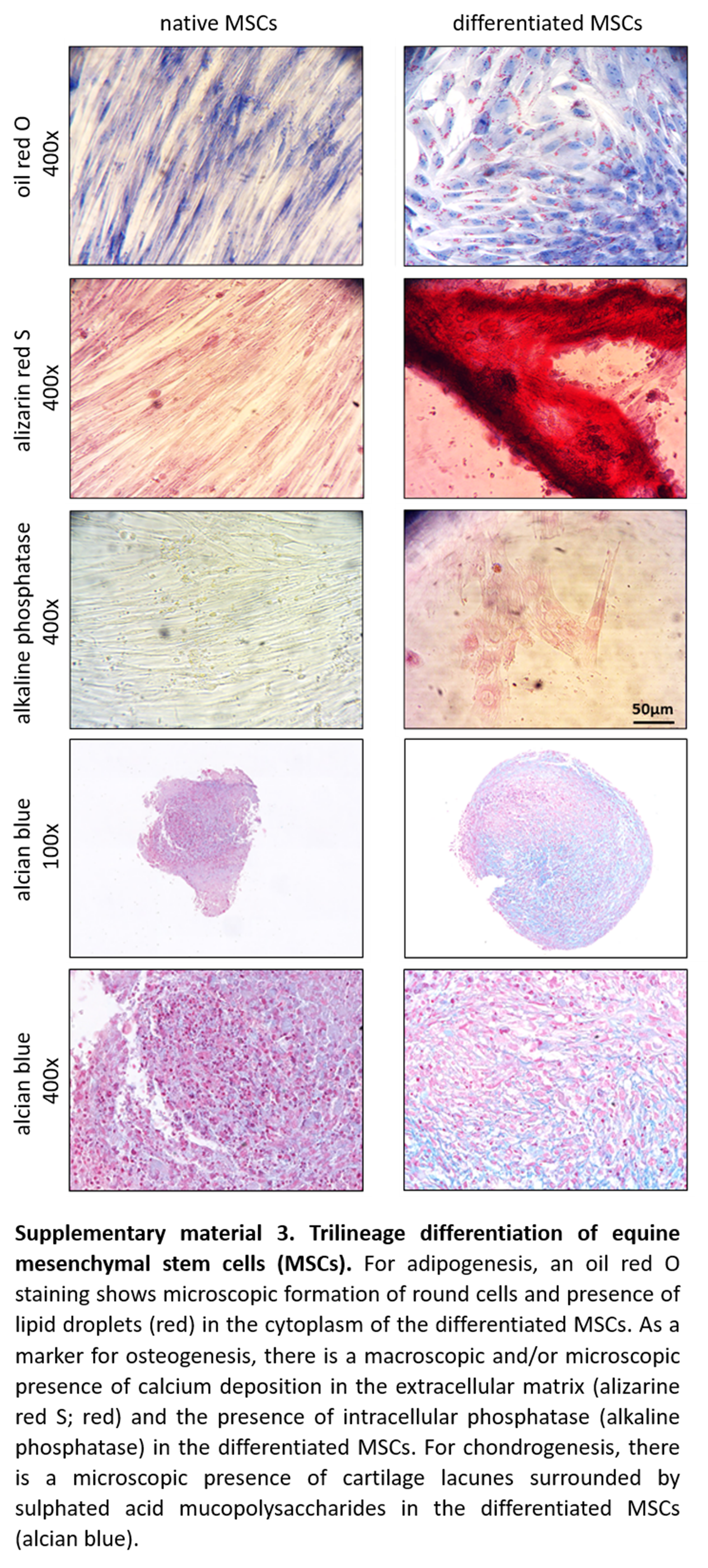

Supplement: Supplementary file 3 [file Image_3.PNG]
